# An *in-vitro* analysis to evaluate the disinfection effectiveness of Cold Atmospheric Pressure (CAP) plasma jet in *Enterococcus faecalis* infected root canals

**DOI:** 10.1080/26415275.2023.2193214

**Published:** 2023-04-21

**Authors:** Pravin Kumar, P. Soundharrajan, Ram Prakash, Sarika Prabhakar Kombade, Pankaj Yadav, Ankita Chugh, Arun Kumar Patnana

**Affiliations:** a Department of Dentistry, All India Institute of Medical Sciences, Jodhpur, India; b Department of Physics, Indian Institute of Technology, Jodhpur, India; c Department of Microbiology, All India Institute of Medical Sciences, Jodhpur, India; d Department of Bioscience and Bioengineering, Indian Institute of Technology, Jodhpur, India; e Department of Dentistry, All India Insitute of Medical Sciences, Rajkot, India

**Keywords:** Endodontics, root canal irrigants, enterococcus faecalis, sodium hypochlorite, cold atmospheric pressure plasma jet, QMix

## Abstract

Cold Atmospheric Pressure (CAP) plasma has shown successful antibacterial efficacy in different medical applications which have prompted researchers to explore its possible use in endodontics. The aim of the present study was to comparatively evaluate the disinfection effectiveness of CAP Plasma jet with 5.25% sodium hypochlorite (NaOCl) and Qmix in *Enterococcus Faecalis* infected root canals at different time intervals (2, 5, and 10 min). 210 single-rooted mandibular premolars were chemomechanically prepared and infected with *E. faecalis*. The test samples were exposed to CAP Plasma jet, 5.25% NaOCl, and Qmix for 2, 5, and 10 min. The residual bacteria from the root canals if any were collected and evaluated for colony-forming units (CFUs) growth. ANOVA and Tukey’s tests were used to evaluate the significant difference between treatment groups. 5.25% NaOCl showed significantly more antibacterial effectiveness (<0.001) when compared with all other test groups except Qmix at 2 and 10 min of exposure time. A minimum contact time of 5 min with 5.25% NaOCl is recommended to get zero bacterial growth in *E. faecalis* infected root canals. QMix requires a minimum contact time of 10 min to achieve optimal CFUs reduction and CAP plasma jet requires a minimum contact time of 5 min to achieve substantial CFUs reduction.

## Introduction

The root canal treatment aims to eliminate the complete polymicrobial infection from the root canal system and achieve a fluid impervious seal [1]. Any failure in achieving the fluid impervious seal, aids in the re-growth of microbes and further results in post-treatment disease. *Enterococcus faecalis* is one of the bacterial species that is known to persist even after routine endodontic treatment [2]. The ability of *E. faecalis* to grow as a biofilm on root canal walls and as a mono-infection in treated canals makes it highly resistant to antimicrobial agents [2].

Sodium hypochlorite (NaOCl) has been used as an endodontic irrigant for more than 70 years. NaOCl is generally a strong oxidizing, hydrolytic agent and it has bactericidal and proteolytic properties. Several studies have reported that NaOCl has wide-spectrum antimicrobial activity and can rapidly kill vegetative and spore-forming bacteria, fungi, protozoa, and viruses [3].

QMix is a new one-step endodontic irrigant containing a mixture of bisbiguanide antibacterial agents (chlorhexidine 2%), calcium chelating agents (ethylenediaminetetraacetic acid) and surfactants [4]. It is also reported that QMix is ​​as effective as 17% ethylenediaminetetraacetic acid in reducing the smear layer [5] and has strong antimicrobial activity. Cold Atmospheric Pressure (CAP) plasma jet applications in medical fields, such as sterilization, promotion of blood coagulation, wound healing, and induction of tumour cell apoptosis, are well proven [6]. CAP plasma has emerged as a novel tool with potential in endodontic root canal disinfection [7].

Though CAP plasma jet has shown promising results in endodontic disinfection its comparison with QMix and NaOCl at different exposure times was not reported yet. Hence, considering the recent developments in irrigation protocols, and lack of adequate literature regarding CAP plasma jet, the present in-vitro study was planned to evaluate the disinfection effectiveness of CAP plasma jet in comparison to sodium hypochlorite and QMix in *E. faecalis* infected root canals at different time intervals. The null hypothesis stated that there would be no difference in the disinfection effectiveness of CAP plasma jet when compared to QMix and NaOCl.

## Materials and methods

The present in-vitro study was conducted at the Department of Dentistry after Institutional ethical clearance (XXXXX/IEC/XXXX/XXXX) in collaboration with the Department of Microbiology at XXXXX XXXXX and the Department of Physics at XXX XXXXX. Two hundred and ten human caries-free single-rooted mandibular premolar teeth with mature apices extracted for orthodontic/periodontal reasons were collected from Departmental OPD.

A digital radiograph was taken for each tooth to either include or exclude according to the criteria followed in the study. Single-rooted mandibular premolar teeth with intact crown and root, mandibular premolars without any carious lesion or defects, teeth with intact and mature root apex, degree of root curvature ≤ 25 degrees, and teeth having a single root canal with single canal orifice and apical foramen were included. Teeth with caries, cracks, endodontic treatments, or restorations, and teeth with any calcification, extra roots and canals, internal and external resorptions, and open apices were excluded from the present study.

### Preparation of the test samples

After scaling with an ultrasonic scaler, teeth were autoclaved and stored in 0.9% normal saline solution until use. The teeth were decoronated below the cementoenamel junction to obtain a standard root length of 12 mm using the diamond discs (DFS, India). All samples were prepared with ProTaper universal hand files (Dentsply, Maillefer, India) up to size F3. The canals were irrigated using 5 ml of 3% NaOCl (SafeEndo Dental India Pvt. LTD) after each instrumentation with a 23-gauge irrigation needle during root canal preparation. The final irrigation was done with 3% NaOCl for 2 min followed by 17% EDTA (Neelkanth Healthcare (P.) LTD, India) for 1 min. The apical foramen of each test sample was sealed with composite resin (3 M, ESPE, India) and the outer surface of the root was coated with nail varnish. The samples were sterilized again in an autoclave for 20 min under 15 psi pressure at 121 °C temperature.

### CAP plasma jet generation

**Figure 1.**
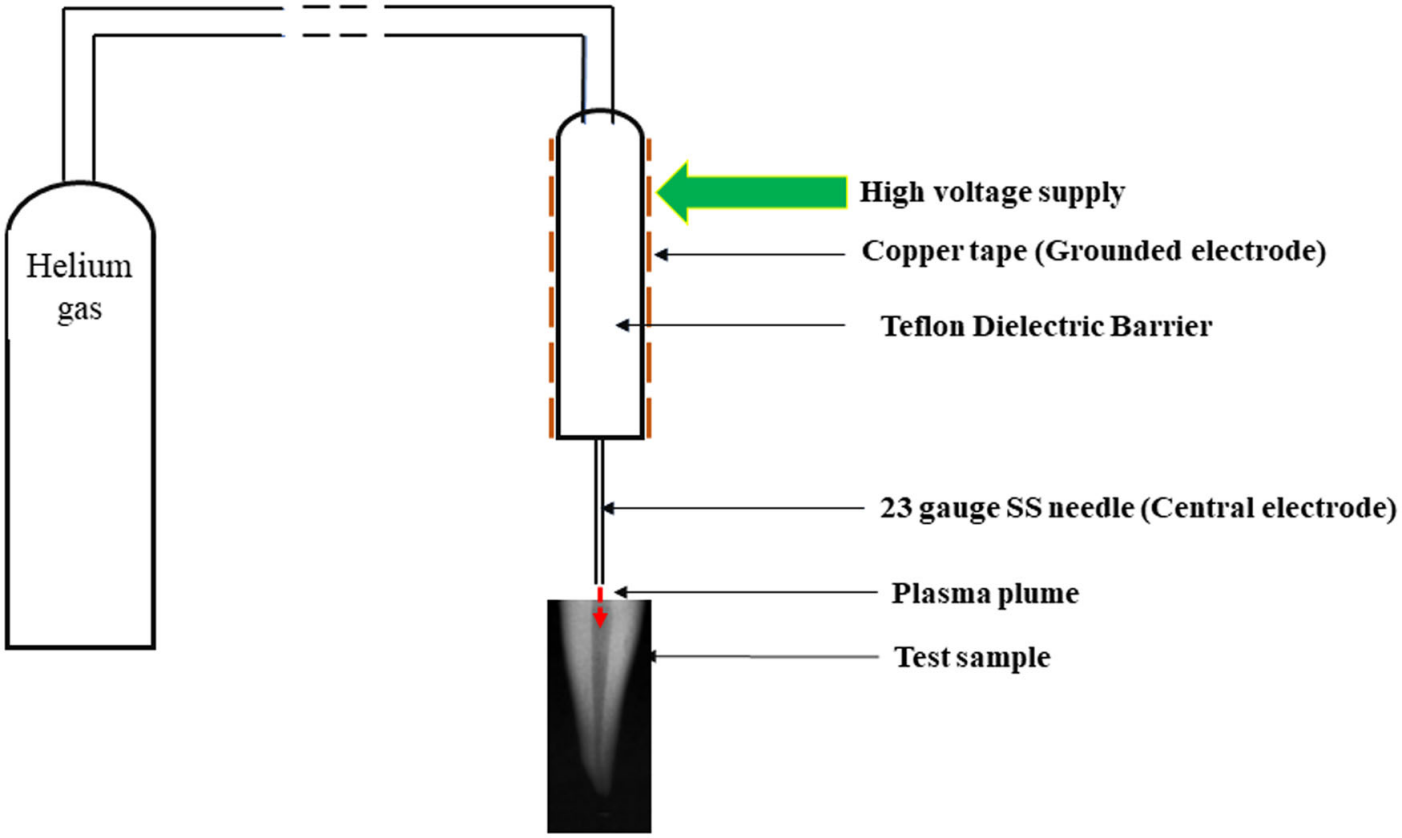
Design of the CAP Plasma jet used in the present study.

**Figure 2.**
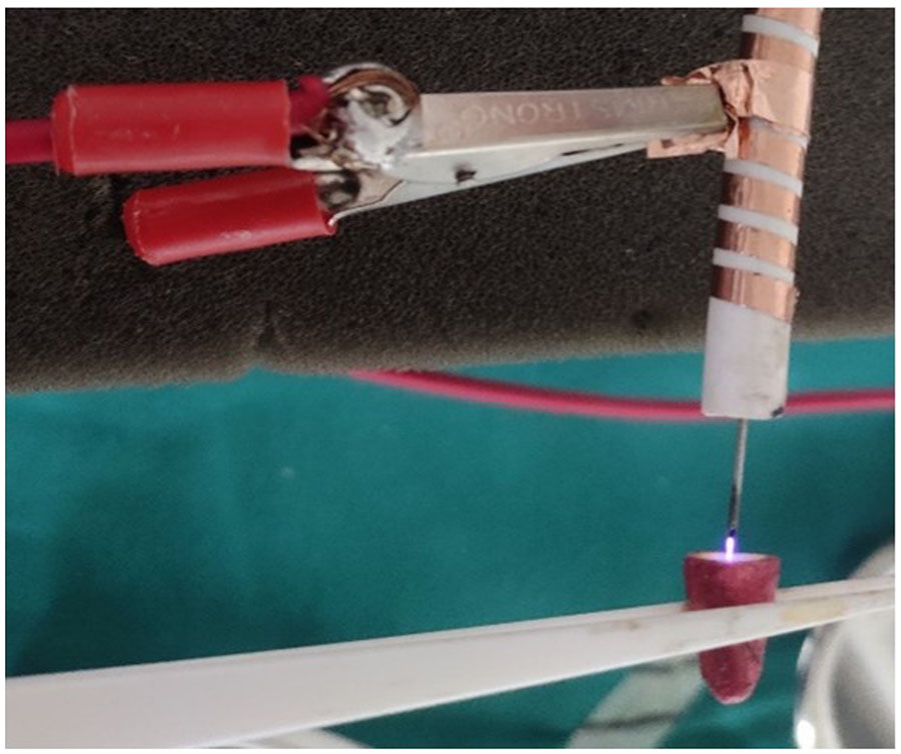
Generation of CAP Plasma jet plume and exposure in the test sample.

A dielectric barrier discharge plasma jet consisting of a SS/copper tube as the central electrode and an axially aligned SS/copper ring as the grounded electrode was taken. A Teflon/Quartz dielectric barrier was used between two electrodes to reduce the flowing current and prevent electrical discharges. The central electrode was connected through a low-power sinusoidal high-voltage source with a 5 kV peak-to-peak voltage. Helium was used as a working gas at a flow rate of 2.5 standard litres per min. The generated CAP plasma plume was exposed *via* a 23-gauge needle (Figures 1 and 2).

### Bacterial culture

The *E. faecalis* (American Type Culture Collection 29212) was cultured in a sterile 50 ml Falcon tube with 30 ml brain-heart infusion (BHI) broth (HI MEDIA, Mumbai, India) at 37 °C. The bacterial concentration used in the experiment was adjusted to 10^5^ colony-forming units (CFUs)/mL. The test samples were divided into three experimental (CAP plasma jet, NaOCl, QMix) groups and one control (0.9% normal saline) group. The experimental groups were further divided into three subgroups (*n* = 21) based on the duration of exposure (2, 5, and 10 min) of the irrigating regimens.

### Experimental root canal infection

The test samples of each subgroup were immersed in 30 ml of BHI broth containing 10^5^ CFUs/mL of *E. faecalis* in falcon tubes. Falcon tubes were placed in Mcintosh anaerobic jar and the samples were incubated anaerobically for 7 days. 5 ml of sterile BHI broth was refreshed every 2 days to ensure the viability of *E.Faecalis*.

### Experimental root canal disinfection

The test samples infected with *E.Faecalis* in each subgroup of the CAP plasma jet were held with a plastic tweezer to orient the coronal orifices of the root canals towards the plasma plume. The samples were then adjusted to direct the plasma plume into the root canal space for respective time durations of 2, 5, and 10 min.

A syringe of 5 ml having a 24-gauge irrigation needle was loaded with 5.25% NaOCl, QMix (Dentsply Tulsa Dental Specialities, US), and Normal saline (Braun group company, India). The needle was inserted 2 mm short of the working length and the solution was expelled slowly to fill the canals in the test samples. Further, NaOCl and QMix were left in the root canals of test samples for different time intervals of 2, 5, and 10 min as per the division of subgroups and normal saline was left in the root canals for 10 min.

### Collection and analysis of the remaining bacteria after disinfection

A sterile barbed broach (Dentsply, Maillefer, India) was inserted into the test samples and churned clockwise and anti-clockwise 10 times to collect the dentinal mud in the root canal space. An F3 paper point (META BIOMED, India) which snugly fitted inside the root canal was used to collect the dentinal mud with residual bacteria if any. This paper point was transferred to the 5 ml falcon tubes (Eppendorf, Mumbai, India) containing 3 ml of BHI broth. The falcon tubes were then shaken for 1 min and 0.25 ml of infected BHI broth was transferred to blood agar plates for bacterial culture. The antimicrobial efficacy was determined by counting the residual bacteria using the CFUs counting method.

### Statistical analysis

A priori sample size analysis was conducted using the PWR2 package in R software to test the difference between ten independent groups. The effect size of 0.4 and 0.2 for the treatment factor and time factor, respectively was used for sample size calculation and a group sample size (N) of 21 samples in each group (total sample size, *N* = 210) was derived.

Statistical analysis was performed using R software (version 4.0.5). At first, the count data measuring bacterial CFUs was log-transformed for further analysis. Next, analysis of variance (ANOVA) and Tukey’s tests was done to determine the significant difference between the various treatment groups. The P-value of less than 0.05 was deemed to be a statistically significant difference. In addition, we fitted Zero-Inflated Poisson (ZIP) model which is considered more suitable for dealing with excess zero-count data.

## Results

### Comparison among treatment groups at fixed time points

Both the ANOVA and the zero-inflated models showed statistically significant (*p* < .05) differences among treatment groups at all three time interval points. At the 2 min time point, 5.25% NaOCl and Qmix showed statistically similar (*p* = .07) and lower CFUs compared with CAP plasma jet (*p* < .001). At 5 min, 5.25% NaOCl showed no presence of CFUs and was thus significantly more effective in CFUs reduction compared with CAP plasma jet and Qmix. Qmix showed significantly lower CFUs than CAP plasma jet (*p* < .001). At 10 min, all three experimental groups (CAP plasma jet, 5.25% NaOCl, and Qmix) showed statistically lower CFUs compared with the control group (*p* < .001), and 5.25% NaOCl and Qmix showed statistically similar (*p* = .545) and lower CFUs (*p* < .001) than the CAP plasma jet **(**Table 1**).**

**Table 1.** Intergroup comparison at fixed time points.

S. No	Time periods	Experimental groups	Mean ± SD of CFU	ANOVA	Zero Inflated Poisson (ZIP) Model	*p* Value (Tukeys test)
1	2 min (*N* = 63)	CAP Plasma Jet (*n* = 21)	2.19 ± 1.16	<0.0001	<0.0001	<.001^a^ <.001^b^ .0753^c^
		5.25 % NaOCl (*n* = 21)	0.09 ± 0.66			
		QMix (*n* = 21)	0.66 ± 0.79			
2	5 min (*N* = 63)	CAP Plasma Jet (*n* = 21)	1.19 ± 0.74	<0.0001	0.005	<.001^a^ .001^b^ .001^c^
		5.25 % NaOCl (*n* = 21)	No CFU (0)			
		QMix (*n* = 21)	0.52 ± 0.67			
3	10 min (*N* = 84)	Control (*n* = 21)	4.76 ± 0.43	<0.0001	<0.0001	<.001^d^ <.001^e^ <.001^f^ <.001^a^ .001^b^ .545^c^
		CAP Plasma Jet (*n* = 21)	0.95 ± 0.66			
		5.25 % NaOCl (*n* = 21)	No CFU (0)			
		QMix (*n* = 21)	0.14 ± 0.35			

^a^Comparison between plasma jet and NaOCl.

^b^Comparison between plasma jet and Qmix.

^c^Comparison between NaOCl and Qmix.

^d^Comparison between normal saline and plasma jet.

^e^comparison between normal saline and NaOCl.

^f^Comparison between normal saline and Qmix.

CAP: Cold Atmospheric Plasma; NaOCl: Sodium Hypochlorite; SD: Standard Deviation; CFU: Colony Forming Units.

### Intra-group comparison at different time points

The Tukey’s test for pairwise comparison of the three groups (CAP Plasma jet, NaOCl, and Qmix) at different time points showed that for the CAP plasma group, results were statistically significant for 2 min *vs.* 5 min and 2 min *vs.* 10 min (Table 2). However, there was no significant difference for the 5 min vs. 10 min comparison (*p* = .662). No significant difference was observed for the NaOCl group between any of the three time points. For the Qmix group, results were statistically significant only for the 2 min *vs.* 10 min comparison (*p* = .026).

**Table 2.** Intra-group comparison at different time points.

S. No	Experimental groups	Subgroups	Mean ± SD of CFU	ANOVA	Zero Inflated Poisson (ZIP) Model	*p* Value (Tukeys test)
1	CAP Plasma Jet (*n* = 63)	2 min (*n* = 21)	2.19 ± 1.16	<0.0001	<0.0001	.001^a^ .0001^b^ .14^c^
		5 min (*n* = 21)	1.19 ± 0.74			
		10 min (*n* = 21)	0.95 ± 0.66			
2	5.25% NaOCl (*n* = 63)	2 min (*n* = 21)	0.09 ± 0.30	0.131	Not Applicable	.13
		5 min (*n* = 21)	No CFU (0)			
		10 min (*n* = 21)	No CFU (0)			
3	QMix (*n* = 63)	2 min (*n* = 21)	0.66 ± 0.79	0.03	0.31	.53^a^ .008^b^ .01^c^
		5 min (*n* = 21)	0.52 ± 0.67			
		10 min (*n* = 21)	0.14 ± 0.35			

^a^Comparison between 2 and 5 min.

^b^Comparison between 2 and 10 min.

^c^Comparison between 5 and 10 min.

CAP: Cold Atmospheric Plasma; NaOCl: Sodium Hypochlorite; SD: Standard Deviation; CFU: Colony Forming Units.

## Discussion

Irrigating solutions and their recent advances have shown that near-zero bacterial counts before obturation can be achieved when suitable concentration and contact time are implemented. CAP plasma jet can reach deep into the infected root canal spaces which are not accessible to the routine irrigating solutions/regimens [8]. It generates reactive oxygen and nitrogen species which either evoke a response on the cell surface or damage intracellular components which eventually results in microbial inactivation [9].

It is reported that the smear layer harbours bacterial colonies which are the reason for post-treatment disease in endodontics. Thus, an additional solution (EDTA) needs to be used for smear layer removal even after the use of NaOCl as an irrigating solution. QMix a relatively new irrigant has become more clinically acceptable as a single irrigating solution with both smear layer removal and anti-bacterial properties which consists of EDTA and CHX [10].

At 2 min of irrigation, 5.25% NaOCl and QMix showed a significant reduction in the CFUs than CAP plasma jet. Though, NaOCl showed the highest bacterial reduction when compared to other experimental regimes none of the experimental solutions yielded complete reduction in the bacterial CFUs. Inability of the experimental irrigating regimes used in the present study to completely eliminate the CFUs from the infected root canals limit the recommendation of 2 min irrigation time.

At 5 min of irrigation, 5.25% NaOCl was the only experimental solution which showed zero growth in the bacterial CFUs. NaOCl acts as an organic tissue and fat solvent, degrading fatty acids and transforming them into fatty acid salts (soap) and glycerol (alcohol), which reduces the surface tension of the solution [11]. When hypochlorous acid, a substance present in NaOCl solution, comes in contact with organic tissue it acts as a solvent and releases chlorine, which combines with the protein amino group to form chloramines. Hypochlorous acid (HOCl^-^) and hypochlorite ions (OCl^-^) lead to amino acid degradation and hydrolysis [3]. The chloramination reaction between chlorine and the amino group (NH) forms chloramines that interfere in cell metabolism. Chlorine (a strong oxidant) has an antimicrobial action, inhibiting bacterial enzymes and leading to an irreversible oxidation of SH groups (sulphydryl group) of essential bacterial enzymes [12]. Thus, the saponification, amino acid neutralization, and chloramination reactions that occur in the presence of microorganisms and organic tissue lead to the antimicrobial effect and tissue dissolution process. NaOCl is a very reactive oxidant that presents a well-documented dissolution and disorganization effect against biofilms [13]. The effectiveness of 5.25% NaOCl against *E. faecalis* observed in the present study was in accordance with the earlier literature [14–16].

In the present study, QMix was as effective as NaOCl in CFUs reduction at two of the three time points. The antibacterial action of QMix can be attributed to its composition which contains CHX and EDTA. The positively charged CHX molecules bind to the negatively charged phospholipids that cause rupture of the bacterial cell wall, which further results in cytoplasmic leakage and cell death [17]. Though QMix has shown a clinically manageable level of CFUs reduction in the present study, it didn’t achieve complete bacterial elimination even at the ten minutes of exposure time.

In our present study, at 10 min of exposure time CAP Plasma jet showed maximum efficiency when compared to other time intervals, but did not result in complete elimination CFUs. The results of the current study contradict the previous reports which showed that 10 min of plasma exposure resulted in the complete elimination of E.Faecalis [18]. This could be due to differences in geometry and in the gases used in the two studies. Previous studies have used Helium/Argon with oxygen as working gases however in the current study Helium was used as the only working gas. He/O_2_ plasma was more effective than He plasma, due to the presence of more reactive oxygen species in He/O_2_ plasma. The lesser reduction in CFUs by the CAP Plasma jet used in the present study may be attributed to this lack of oxygen utilisation as part of its geometry. To improve the antibacterial effectiveness of the CAP Plasma jet modification in the following factors jet length, jet power, jet volume, and flow rate of the gases can be considered.

Though CFUs method of *E. faecalis* detection is considered traditional and gold standard, newer detection methods such as Polymerase chain reaction (PCR) and PCR-based technologies (real-time PCR and multiplex PCR) may be considered for rapid, specific and sensitive results in future studies.

## Conclusion

At all time intervals of 2,5 and 10 min, 5.25% NaOCl is most effective in CFUs reduction followed by Qmix and CAP plasma jet. A minimum exposure time of 5 min is required for complete elimination of CFUs with 5.25% NaOCl. A minimum time interval of 10 min is required to achieve optimal CFUs reduction with Qmix. A minimum exposure time of 5 min is required to achieve substantial CFUs reduction with CAP plasma jet and at 10 min of time interval better results were observed. Irrigation with 5.25% NaOCl for a minimum time period of 5 min may be considered as the preferred irrigating regime considering complete elimination of CFUs in the *E.faecalis* infected root canals.

## References

[1] Somani R, Jaidka S, Singh DJ, et al. Hermetic seal in obturation: an achievable goal with recently introduced cpoint. Int J Clin Pediatr Dent. 2019;12(5):410–413. 32440046 10.5005/jp-journals-10005-1619 PMC7229356

[2] Sakamoto M, Siqueira JF, Rôças IN, et al. Bacterial reduction and persistence after endodontic treatment procedures. Oral Microbiol Immunol. 2007;22(1):19–23. 17241166 10.1111/j.1399-302X.2007.00315.x

[3] Mohammadi Z. SS. Antimicrobial activity of sodium hypochlorite in endodontics. J Mass Dent Soc Spring. 2013;62(1):28–31. 24494267

[4] Arslan D, Guneser MB, Kustarci A, et al. Pulp tissue dissolution capacity of QMix 2in1 irrigation solution. Eur J Dent. 2015;9(3):423–427. 26430374 10.4103/1305-7456.163229 PMC4569997

[5] Dai L, Khechen K, Khan S, et al. The effect of QMix, an experimental antibacterial root canal irrigant, on removal of canal wall smear layer and debris. J Endod. 2011;37(1):80–84. 21146083 10.1016/j.joen.2010.10.004

[6] Braný D, Dvorská D, Halašová E, et al. Cold atmospheric plasma: a powerful tool for modern medicine. IJMS. 2020;21(8):2932. 32331263 10.3390/ijms21082932 PMC7215620

[7] Lu X, Cao Y, Yang P, et al. An RC plasma device for sterilization of root canal of teeth. IEEE Trans Plasma Sci. 2009;37(5):668–673.

[8] Du T, Ma J, Yang P, et al. Evaluation of antibacterial effects by atmospheric pressure nonequilibrium plasmas against Enterococcus faecalis biofilms in vitro. J Endod. 2012;38(4):545–549.. 22414847 10.1016/j.joen.2011.10.021

[9] Puligundla P, Mok C. Inactivation of spores by nonthermal plasmas. World J Microbiol Biotechnol. 2018;34(10):143. 30203172 10.1007/s11274-018-2527-3

[10] Lim BSH, Parolia A, Chia MSY, et al. Antimicrobial efficacy of QMix on Enterococcus faecalis infected root canals: a systematic review of in vitro studies. Restor Dent Endod. 2020;45(2):e23. 32483540 10.5395/rde.2020.45.e23 PMC7239686

[11] Haapasalo M, Shen Y, Wang Z, et al. Irrigation in endodontics. Br Dent J. 2014;216(6):299–303. 24651335 10.1038/sj.bdj.2014.204

[12] Zehnder M. Root canal irrigants. J Endod. 2006;32(5):389–398. 16631834 10.1016/j.joen.2005.09.014

[13] Alves FRF, Almeida BM, Neves MAS, et al. Time-dependent antibacterial effects of the self-adjusting file used with two sodium hypochlorite concentrations. J Endod. 2011;37(10):1451–1455. 21924201 10.1016/j.joen.2011.06.001

[14] Giardino L, Ambu E, Savoldi E, et al. Comparative evaluation of antimicrobial efficacy of sodium hypochlorite, MTAD, and tetraclean against Enterococcus faecalis biofilm. J Endod. 2007;33(7):852–855. 17804328 10.1016/j.joen.2007.02.012

[15] Schaudinn C, Jaramillo D, Freire MO, et al. Evaluation of a nonthermal plasma needle to eliminate ex vivo biofilms in root canals of extracted human teeth. Int Endod J. 2013;46(10):930–937. 23480318 10.1111/iej.12083 PMC3687033

[16] Ballout H, Hertel M, Doehring J, et al. Effects of plasma jet, dielectric barrier discharge, photodynamic therapy and sodium hypochlorite on infected curved root canals. J Biophotonics. 2018;11(3):e201700186. 10.1002/jbio.201700186 29024574

[17] Stojicic S, Shen Y, Qian W, et al. Antibacterial and smear layer removal ability of a novel irrigant, QMiX. Int Endod J. 2012;45(4):363–371. 23134158 10.1111/j.1365-2591.2011.01985.x

[18] Pan J, Sun K, Liang Y, et al. Cold plasma therapy of a tooth root canal infected with enterococcus faecalis biofilms in vitro. J Endod. 2013;39(1):105–110. 23228267 10.1016/j.joen.2012.08.017

